# The relationship between aspirin consumption and hepatocellular carcinoma: a systematic review and meta-analysis

**DOI:** 10.1186/s40001-023-01204-5

**Published:** 2023-07-08

**Authors:** Shuai Wang, Lijuan Zuo, Zhaojin Lin, Zhiqin Yang, Ran Chen, Yan Xu

**Affiliations:** 1 Department of Gastroenterology, China-Japan Union Hospital of Jilin University, No. 126 Xiantai Street, Changchun, 130033 Jilin China; 2 Department of Gastroenterology, The First Affiliated Hospital of Weifang Medical University, Weifang, China; 3 Department of Anesthesiology, The First Affiliated Hospital of Weifang Medical University, Weifang, China

**Keywords:** Aspirin, Hepatocellular carcinoma, Meta-analysis

## Abstract

**Background:**

Recent studies have shown that aspirin consumption may reduce the risk of hepatocellular carcinoma (HCC), but their correlation is still not fully understood. This meta-analysis aimed to investigate the correlation between aspirin consumption and HCC.

**Methods:**

A systematic literature search was conducted on PubMed, Scopus, Cochrane Library, EMBASE, and Web of Science databases. The search period was from the establishment of the database to July 1, 2022 with no language restrictions.

**Results:**

A total of 19 studies including three prospective studies and 16 retrospective ones with 2,217,712 patients were included. Compared with those who did not take aspirin, those who took aspirin had a 30% lower risk of HCC (hazard ratio [HR] = 0.70, 95% confidence interval [CI] 0.63–0.76, *I*^2^ = 84.7%, *P* < 0.001). Subgroup analysis showed that aspirin significantly reduced the risk of HCC by 19% in Asia (HR = 0.81, 95% CI 0.80–0.82, *I*^2^ = 85.2%, *P* < 0.001) and by 33% (HR = 0.67, 95% CI 0.61–0.73, *I*^2^ = 43.6%, *P* = 0.150) in Europe and the U.S with no significant difference. Moreover, in patients with HBV or HCV infection, aspirin reduced 19% and 24% of the risk of HCC, respectively. However, aspirin administration might increase risks of gastrointestinal bleeding in patients with chronic liver disease (HR = 1.14, 95% CI 0.99–1.31, *I*^2^ = 0.0%, *P* = 0.712). Sensitivity analysis showed no significant difference of results after excluding individual studies, suggesting that the results were robust.

**Conclusion:**

Aspirin may reduce the risk of HCC in both healthy population and patients with chronic liver disease. However, attention should be paid to adverse events such as gastrointestinal bleeding in patients with chronic liver disease.

**Supplementary Information:**

The online version contains supplementary material available at 10.1186/s40001-023-01204-5.

## Introduction

Hepatocellular carcinoma (HCC) is the most common primary liver malignancy that accounts for 75–85% of liver cancers. Patients with HCC have poor prognoses and the onset of HCC is insidious. Risk factors of HCC include chronic liver inflammation and liver fibrosis or cirrhosis. In recent years, the global incidence of HCC has increased and it is estimated that more than 1 million patients will die from HCC in 2030.

Most HCC patients don’t have the opportunity for surgery because of advanced stages. Only a small number of patients that are diagnosed at an early stage can receive radical resection [2]. Other therapies such as transcatheter arterial chemoembolization, targeted therapy, or immunotherapy also had limited efficacy [3]. Therefore, there is an urgent need for effective therapies to control the progression of HCC.

Aspirin is a classical non-steroidal anti-inflammatory drug (NSAIDs) that exerts antiplatelet effects by acetylating platelet cyclooxygenase (COX), thereby reducing the risk of myocardial infarction and stroke [4]. Therefore, it is widely used in the prevention and treatment of cardiovascular diseases. In the last few decades, much evidence has shown the potential of aspirin in the prevention and treatment of cancer [5–7]. A randomized trial including 25,570 participants showed that aspirin reduced the risk of death from gastrointestinal cancers, including esophageal, colorectal, and pancreatic cancers, as well as from non-gastrointestinal solid cancers, such as lung, prostate, bladder, and kidney cancers, especially colorectal cancers [8]. Studies have demonstrated that aspirin can reduce the risk of HCC, whereas non-aspirin NSAID does not have this effect [9, 10]. Moreover, compared with irregular aspirin administration, the risk-reducing effect of aspirin is dose- and duration dependent [6]. In in vitro and in vivo experiments, the combination of aspirin and other drugs exhibited a promising efficacy to suppress HCC progression [11–14].

However, a randomized controlled trial showed that long-term administration of low-dose aspirin did not reduce the risk of overall cancer, breast cancer, colorectal cancer, or other site-specific cancers [15]. Therefore, it remains controversial whether aspirin can reduce the risk of HCC. This study aimed to investigate the association between aspirin use and HCC through a meta-analysis.

## Materials and methods

### Literature retrieval strategy

A systematic literature search was conducted on PubMed, Scopus, Cochrane Library, EMBASE and Web of Science databases from the establishment of the database to July 1, 2022, with no language restrictions. A combination of subject terms and free words were used for literature search using Boolean logic operator grouping (Additional file 1).

### Inclusion and exclusion criteria

Inclusion criteria were as follows: (1) retrospective or prospective cohort studies that explored the relationship between aspirin and the risk of HCC (from 2015 to 2022); (2) reporting the hazard ratio (HR) and 95% confidence interval (CI) of HCC based on different administration of aspirin; (3) the diagnosis of HCC was made based on the diagnostic criteria. Exclusion criteria were as follows: (1) reviews, case reports, meta-analyses, conference abstracts, redundant publications, ecological studies, animal studies, and case–control studies; (2) studies on patients who have been diagnosed with HCC before aspirin use; (3) studies that did not explicitly report any HR or 95% CI.

### Evaluation of literature quality

The Newcastle–Ottawa scale (NOS) was used to evaluate literature quality in terms of study population selection, comparability between groups, and outcome measures, with a maximum score of 9. We included high-quality literature with scores greater than or equal to 7 in this study.

### Data extraction

Two investigators independently screened the literature and extracted data according to the criteria. In case of disagreements, they discussed or consulted a third party to reach the consensus. During literature screening, the title was first read to exclude irrelevant literature, then the abstract and full text were read to include relevant studies. Corresponding authors were contacted by email to obtain necessary information if needed. The extracted information included (1) first author, year of publication, study region, and study type; (2) study population and whether they had liver disease; (3) the number of participants and cases; (4) reported outcomes, including HR and 95% CI and (5) study-adjusted confounding factors, etc.

### Statistical analysis

Stata 14.2 software was used for meta-analysis, and the statistics were presented by HR and 95% CI, with a preference for the corrected values if they were provided in the literature. The Cochran q test (P heterogeneity) and the *I*^2^ value were used to evaluate the heterogeneity of the included studies. *P* ≥ 0.05, 0 ≤ *I*^2^ ≤ 50% indicated low heterogeneity, and a fixed effect model was used for analysis; *P* < 0.05 and *I*^2^ > 50% indicated significant heterogeneity, so that a random effect model was used to identify the source of heterogeneity and perform subgroup and sensitivity analyses. Publication bias was evaluated using a funnel plot, Beggs test, and Eggers. *P* < 0.05 indicated that the difference was statistically significant.

## Results

### Literature screening process

**Fig. 1:**
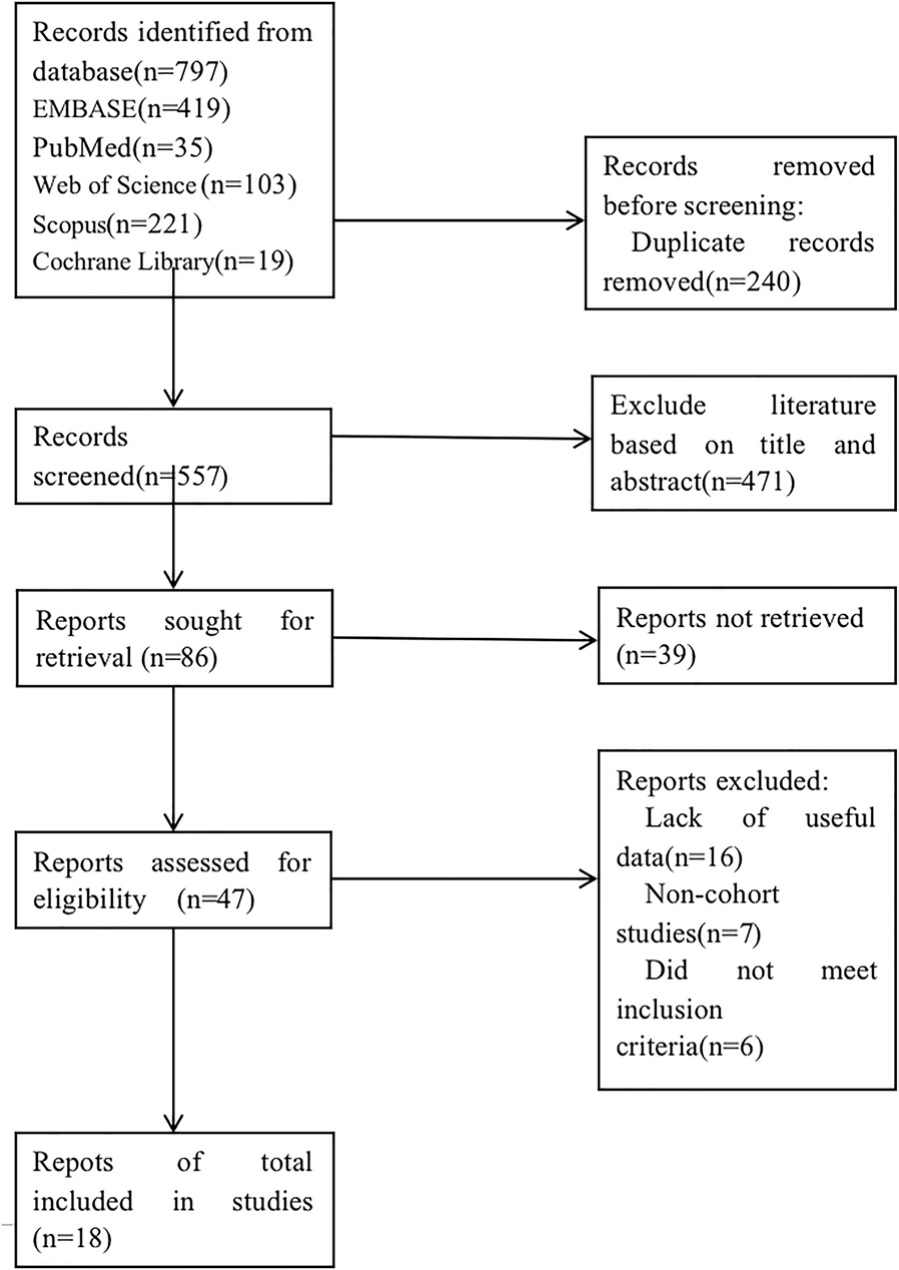
Literature selection process

We retrieved 35, 221, 103, 419, and 19 papers from Pubmed, Scopus, Web of Science, EMBASE, and Cochrane Library, respectively. 240 duplicate papers were excluded. After reading the titles and abstracts and excluding irrelevant literature such as reviews, case reports, meta-analyses, conference abstracts, animal experimental studies, 49 articles were selected for full-text reading. Finally, 18 articles with a total of 2,217,712 participants were enrolled based on the criteria. The literature screening process is shown in Fig. 1.

### Basic characteristics and quality evaluation of the included literature

This article included 18 articles containing 19 studies published from 2015 to 2022, in which 13 studies focused on patients with liver disease, 4 studies focused on general population, and the remaining 2 studies focused on specific patients with type 2 diabetes or head and neck squamous cell carcinoma (HNSCC); 3 were prospective studies and 16 were retrospective studies (Table 1). Based on NOS results, all included literature had high quality (Table 2).

**Table 1:** Basics features of the included literature

Author	Year	Region	Type of Research	Number of participants	Number of cases	Study population	HR(95% CI)	Confounding factors for adjustment
Yun B [16]	2022	Korea	RC	161673	7083	HBV-infected patients	0.83 (0.75–0.93)	Age, sex, hypertension, diabetes mellitus, dyslipidemia, cirrhosis, antivirals, metformin, statin, smoking, alcohol consumption, and obesity
Singh J [17]	2022	USA	RC	521	45	Liver cirrhosis	0.266 (0.094–0.755)	Age, sex, and CTP in the multivariable model
Won- Mook Choi [18]	2021	Korea	RC	32695	6539	HBV-infected patients	0.81 (0.80–0.82)	Age, gender, socioeconomic status, diabetes mellitus, hypertension, smoking, alcohol consumption, BMI, ALT, and combination medication
Vicki Wing-Ki Hu [19]	2021	Hong Kong	RC	35111	1557	HBV-infected patients	0.60 (0.46–0.78)	Age, gender, cirrhosis, hypertension, renal replacement therapy, creatinine, diabetes mellitus, platelet, albumin, total bilirubin, ALT, HBeAg +
Simon,T.G [20]	2020	Sweden	PC	50275	1612	HBV and HCV infected patients	0.69 (0.62–0.76)	Gender, consecutive years since diagnosis of hepatitis B or C, severity of liver disease, use of antiviral therapy, presence or absence of diabetes, hypertension, obesity or alcohol abuse, misuse, and use of insulin, metformin and statins
Shin,S [21]	2020	Korea	RC	949	133	Alcoholic cirrhosis	0.13 (0.08–0.21)	Age, gender, Child–Pugh score, MELD score, AST, ALT, albumin, total bilirubin, creatinine, INR and platelet count
Liao YH [22]	2020	Taiwan	RC	3822	278	HCV- infected patients	0.56 (0.43–0.72)	Gender, age, hypertension, diabetes, moderate to severe liver disease, myocardial infarction, congestive heart failure, ischemic stroke, antihypertensives, hypoglycemic agents, coumarins and heparins, other antithrombotic drugs and NSAIDs
Lee,T.Y [23]	2020	Taiwan	RC	7434	436	HCV- infected patients	0.78 (0.64–0.95)	Age, gender, cirrhosis, hepatic decompensation, hyperlipidemia, statin use and interferon therapy
Sung JJ [24]	2020	Hong Kong	RC	138966	751	General population	0.66 (0.59–0.74)	Age, gender, other medications (including H2 antagonists, statins, NSAIDs, and anticoagulants), comorbidities (coronary artery disease, stroke)
Lee,T.Y [25]	2019	Taiwan	RC	10615	697	HBV-infected patients	0.71 (0.58–0.86)	Age, gender, cirrhosis, diabetes, hyperlipidemia, hypertension, statin use, metformin use, and nucleic acid analogue use
Simon,T.G [6]	2018	USA	PC	133371	108	General population	0.51 (0.34–0.77)	Gender, age, race/ethnicity, body mass index, alcohol consumption, smoking status, physical activity, diabetes, hypertension, dyslipidemia, regular use of multivitamins, antidiabetic drugs, statins, routine use of non-aspirin NSAIDs
Hwang,I.C [26]	2018	Korea	RC	460755	2336	General population	0.87 (0.77–0.98)	Age, sex, body mass index, smoking, alcohol consumption, physical activity, concomitant medication, blood pressure category, fasting glucose and total cholesterol, socioeconomic status, and CCI
Ho CM- HBV [27]	2018	Taiwan	RC	7724	552	HBV-infected patients	0.82 (0.67–1.01)	Gender, age, low income, cirrhosis, diabetes, hyperlipidemia, malignancies other than hepatocellular carcinoma, COPD, ESRD, transplantation, alcohol consumption, and concomitant use of ACEIs/ARBs, metformin, and statins
Ho CM- HCV [27]	2018	Taiwan	RC	7873	503	HCV- infected patients	0.55 (0.23–1.28)	Gender, age, low income, cirrhosis, diabetes, hyperlipidemia, malignancies other than hepatocellular carcinoma, COPD, ESRD, transplantation, alcohol consumption, and concomitant use of ACEIs/ARBs, metformin, and statins
Tseng CH [28]	2018	Taiwan	RC	43800	1750	Patients with type 2 diabetes	0.83 (0.69–0.99)	Age, sex, occupation and area of residence, major comorbidities (hypertension, dyslipidemia and obesity), diabetes-related complications, use of antidiabetic medications (insulin, sulfonylureas, meglitinides, acarbose, rosiglitazone and pioglitazone), potential risk factors for cancer (chronic obstructive pulmonary disease, tobacco abuse, alcohol-related diagnoses, gallstones, history of Helicobacter pylori infection, EB virus-related diagnoses, hepatitis B virus infection, hepatitis C virus infection, cirrhosis and other chronic non-alcoholic liver disease) and medications commonly used or that may affect cancer risk in patients with diabetes (ACEI/ARB, calcium channel blockers, statins, betablockers and aspirin)

Lin YS [29]	2018	Taiwan	RC	18243	110	HNSCC	0.67 (0.42–1.08)	Age, gender, urbanization, coronary artery disease, hypertension, diabetes, atrial fibrillation, heart failure, hyperlipidemia, chronic kidney disease, and COX2 and statin use
Lee, M [30]	2017	Korea	RC	1674	63	HBV-infected patients	0.28 (0.11–0.75)	Age, sex, diabetes, cirrhosis, Child–Pugh score, MELD score, HBeAg, ALT, albumin, total bilirubin, Scr, PT and platelet count
Lee, T.Y [31]	2017	Taiwan	RC	18080	41	NAFLD	0.70 (0.37–1.36)	Age, gender, ALT elevation, hypertension, hypercholesterolemia, diabetes, gout, statin use, metformin use
Petrick, J.L [32]	2015	USA	PC	1084133	679	General population	0.63 (0.50–0.78)	Gender, age, race, cohort, BMI, smoking status, alcohol consumption

*HCC* hepatocellular carcinoma, *TACE* transcatheter arterial chemoembolization, *NSAIDs*, nonsteroidal anti-inflammatory drug, *COX* cyclooxygenase, *MI* myocardial infarction, *NOS* Newcastle–Ottawa scale, *HNSCC* head and neck squamous cell carcinoma, *PC* prospective cohort, *RC* retrospective cohort, *HR* hazard ratio, *BMI* body mass index, AST aspartate aminotransferase, *ALT* alanine aminotransferase, INR international normalized ratio, *PT* prothrombin time, *MELD* Model for End-Stage Liver Disease, *CCI* Chronic Coronary Insufficiency, *COPD* chronic obstructive pulmonary disease, *ESRD* end-stage renal disease

**Table 2:** Literature quality evaluation

Inclusion in the literature	Crowd selection	Comparability between group	Measurement results	NOS score
Yun B [16]	4	2	3	9
Singh J [17]	4	1	3	8
Won- Mook Choi [18]	4	2	2	8
Vicki Wing-Ki Hui [19]	4	2	3	9
Simon, T.G. [20]	4	2	3	9
Shin, S [21]	4	1	3	8
Liao YH [22]	4	2	3	9
Lee, T.Y. [23]	4	2	3	9
Sung JJ [24]	4	2	3	9
Lee, T.Y. [25]	4	2	3	9
Simon, T.G. [6]	4	2	3	9
Hwang, I.C. [26]	4	2	3	9
Ho CM-HBV [27]	4	2	3	9
Ho CM-HCV [27]	4	2	3	9
Tseng CH [28]	4	2	3	9
Lin YS [29]	3	2	2	7
Lee, M [30]	4	1	3	8
Lee, T.Y. [31]	4	2	3	9
Petrick, J.L. [32]	4	2	3	9

### Aspirin was associated with reduced risk of HCC

**Fig. 2:**
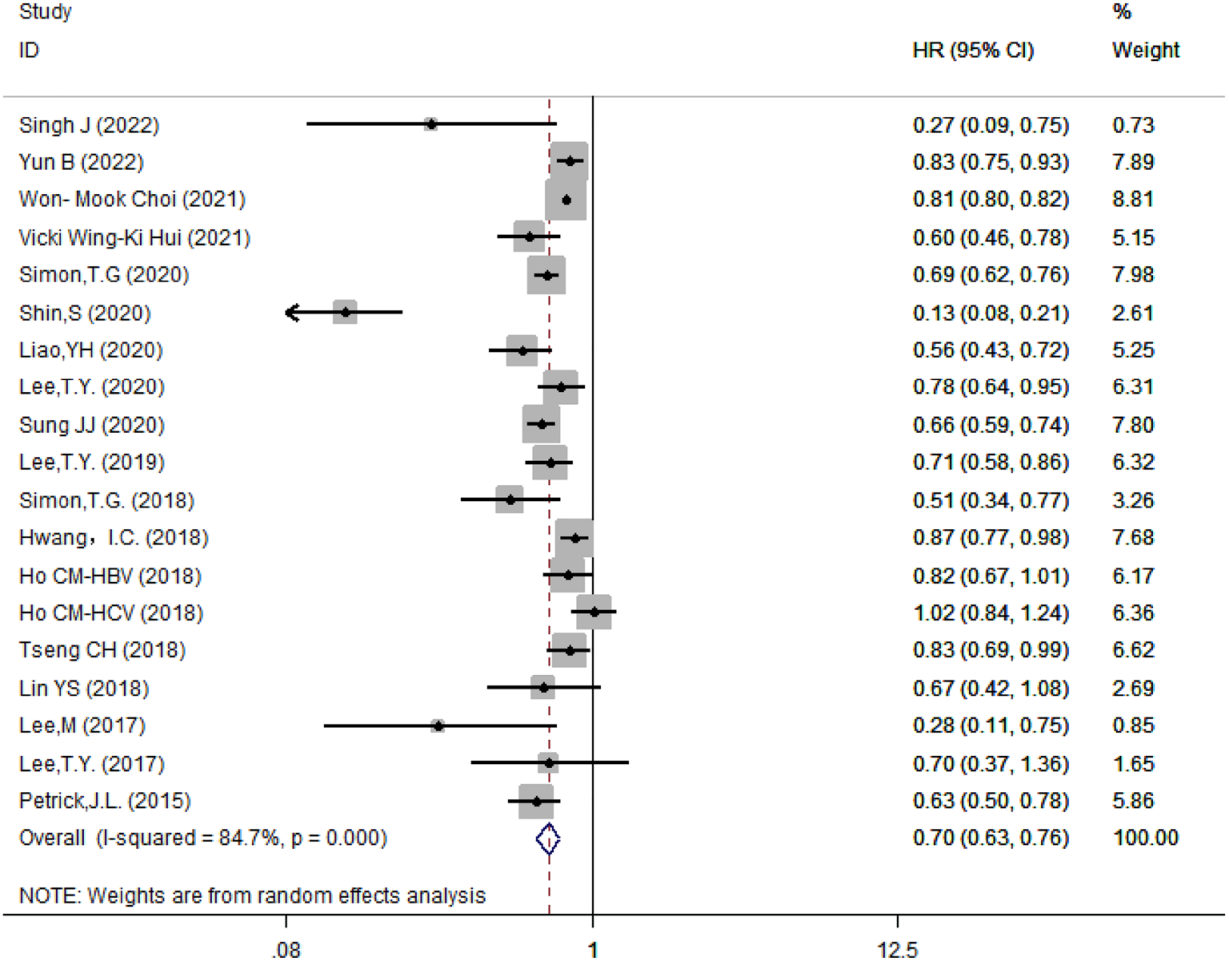
Forest plot of correlation between aspirin and the risk of hepatocellular carcinoma

**Fig. 3:**
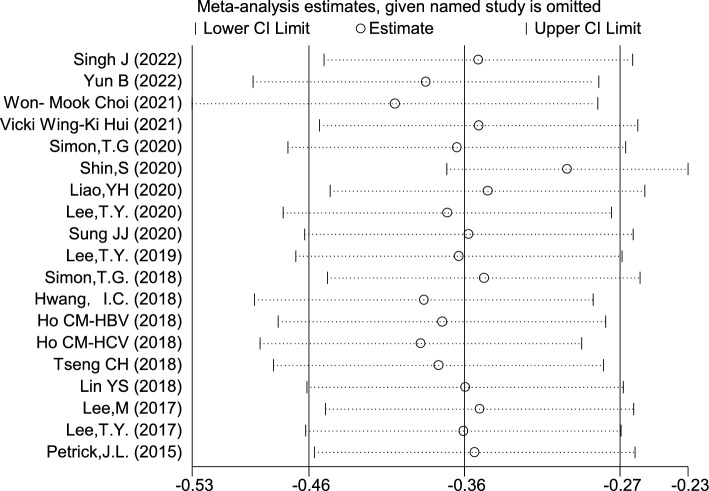
Sensitivity analysis results

The 19 included studies showed a high heterogeneity, therefore, random effect model was adopted and showed that the risk of HCC was 30% lower in those taking aspirin compared with those not taking aspirin (HR = 0.70, 95% CI 0.63–0.76, *I*^2^ = 84.7%, *P* < 0.001) (Fig. 2). The results showed that there was no significant difference of HR after excluding individual studies (Fig. 3), suggesting that the results were robust.

### Subgroup analysis of the association between aspirin and the risk of HCC

#### Subgroup analysis based on regions

**Fig. 4:**
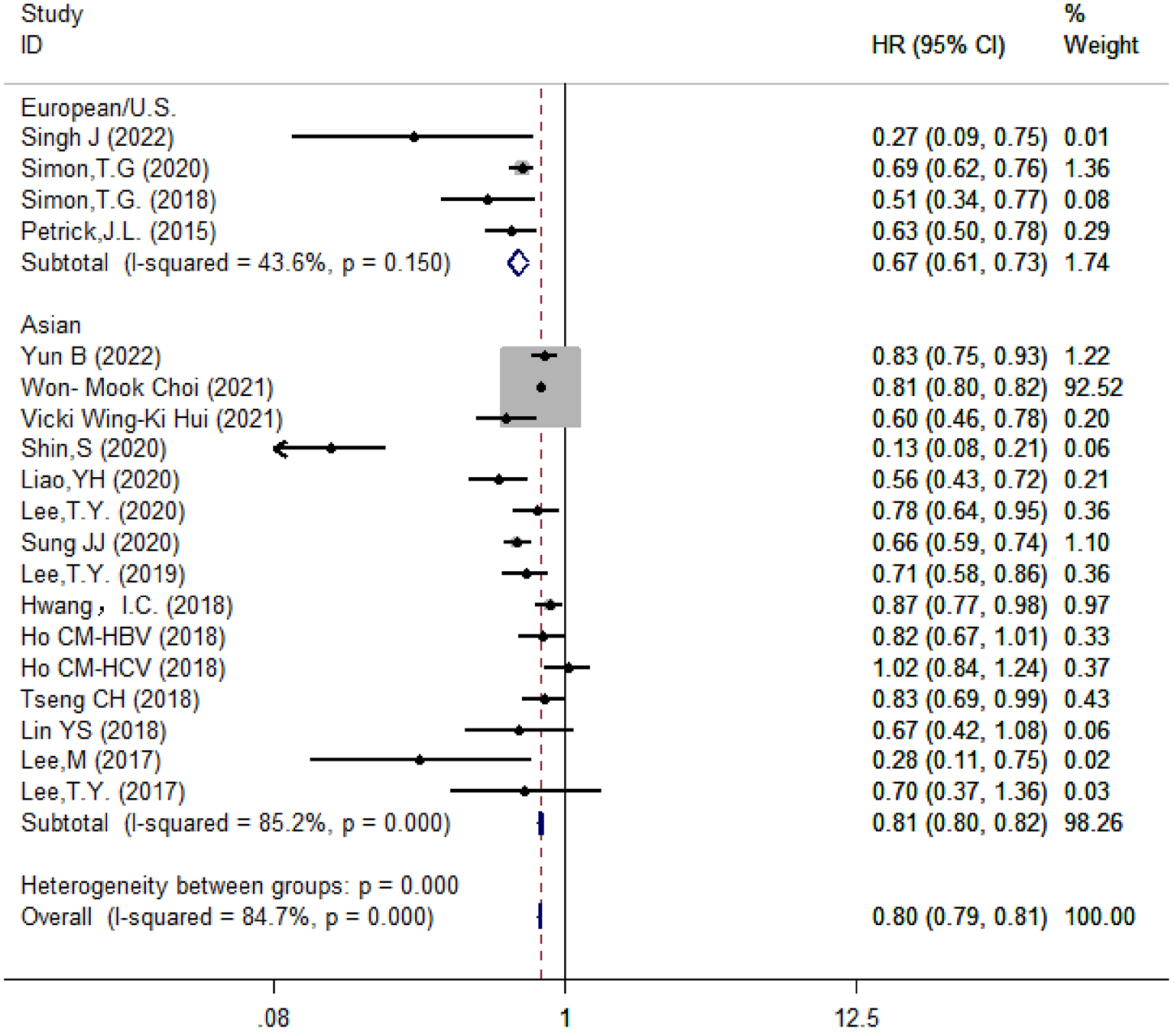
Subgroup analysis of forest plots based on regions

Since aspirin and the risk of HCC. The studies were divided into two subgroups based on regions: the Asia group contained 15 studies whereas the Europe and the United States group had 4 studies. The results showed that aspirin reduced the risk of HCC by 19% in Asia (HR = 0.81, 95% CI 0.80–0.82, *I*^2^ = 85.2%, *P* < 0.001) and by 33% in Europe and the U.S. (HR = 0.67, 95% CI 0.61–0.73, *I*^2^ = 43.6%, *P* = 0.150) (Fig. 4).

#### Subgroup analysis based on study design

**Fig. 5:**
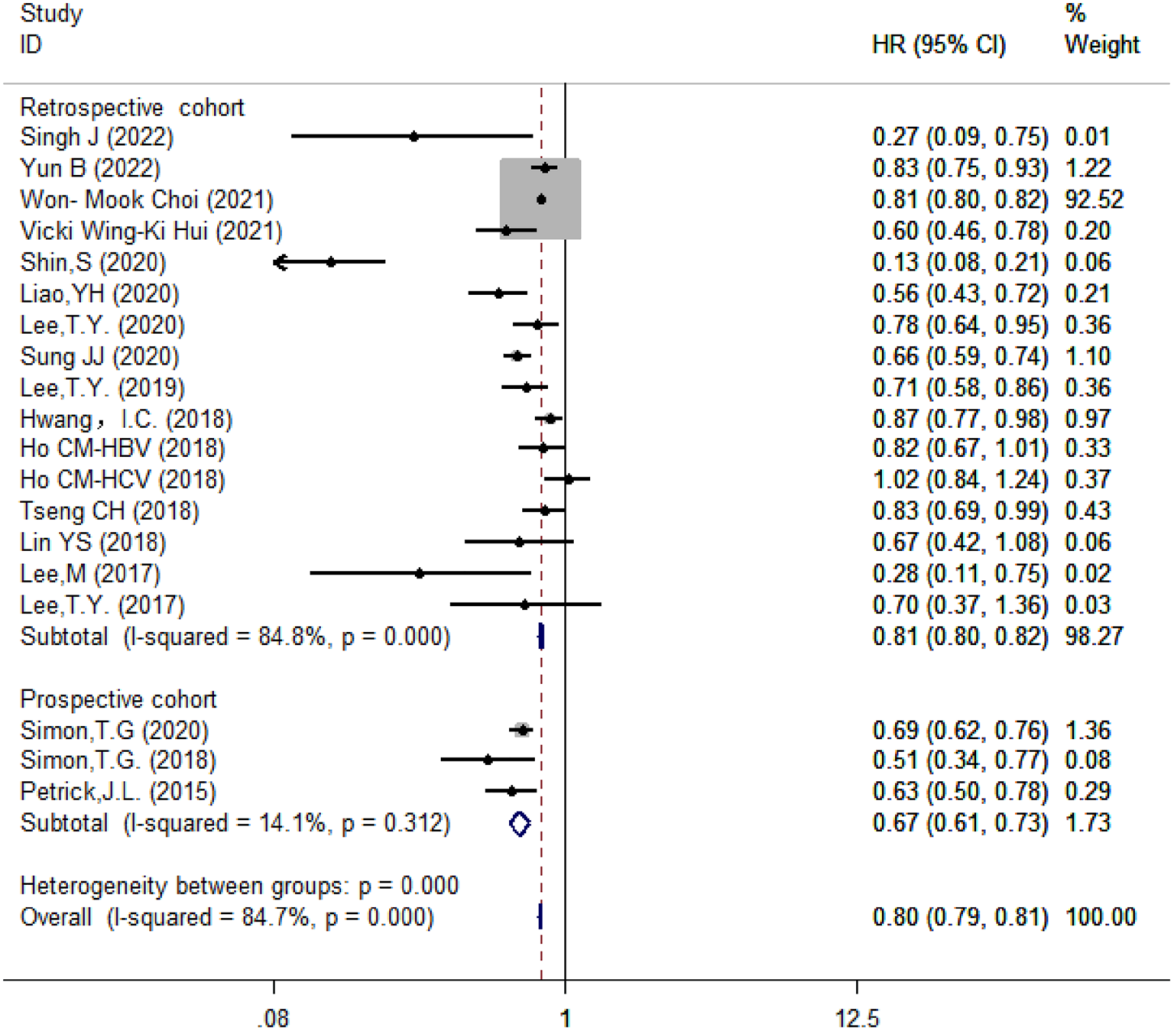
Subgroup analysis of forest plots based on study design

Moreover, based on study design, these studies were classified into prospective (*n* = 3) and retrospective (*n* = 16) studies. The results showed that aspirin reduced the risk of HCC in the retrospective studies (HR = 0.81, 95% CI 0.80–0.82, *I*^2^ = 84.8%, *P* < 0.001) (Fig. 5).

#### Subgroup analysis based on gender

**Fig. 6:**
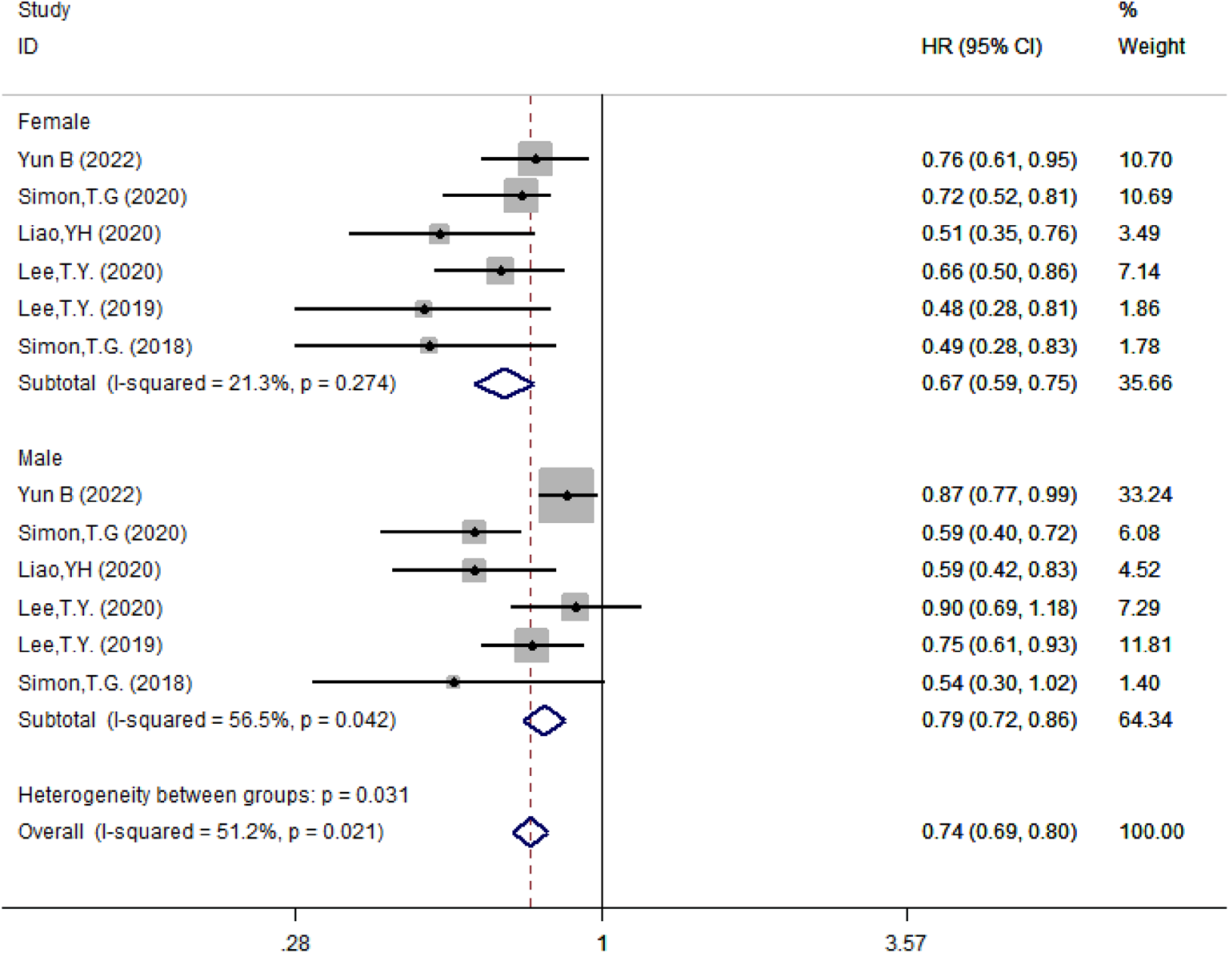
Subgroup analysis of forest plots based on gender

According to genders, studies were divided into female and male groups, with a total of five papers reporting the HR for both female and male. The results showed that aspirin reduced the risk of HCC by 33% in female (HR = 0.67, 95% CI 0.59–0.75, *I*^2^ = 21.3%, *P* = 0.274) and by 21% in male (HR = 0.79, 95% CI 0.72–0.86, *I*^2^ = 56.5%, *P* = 0.042) (Fig. 6).

#### Subgroup analysis based on whether results were adjusted by statins and metformin

**Fig. 7:**
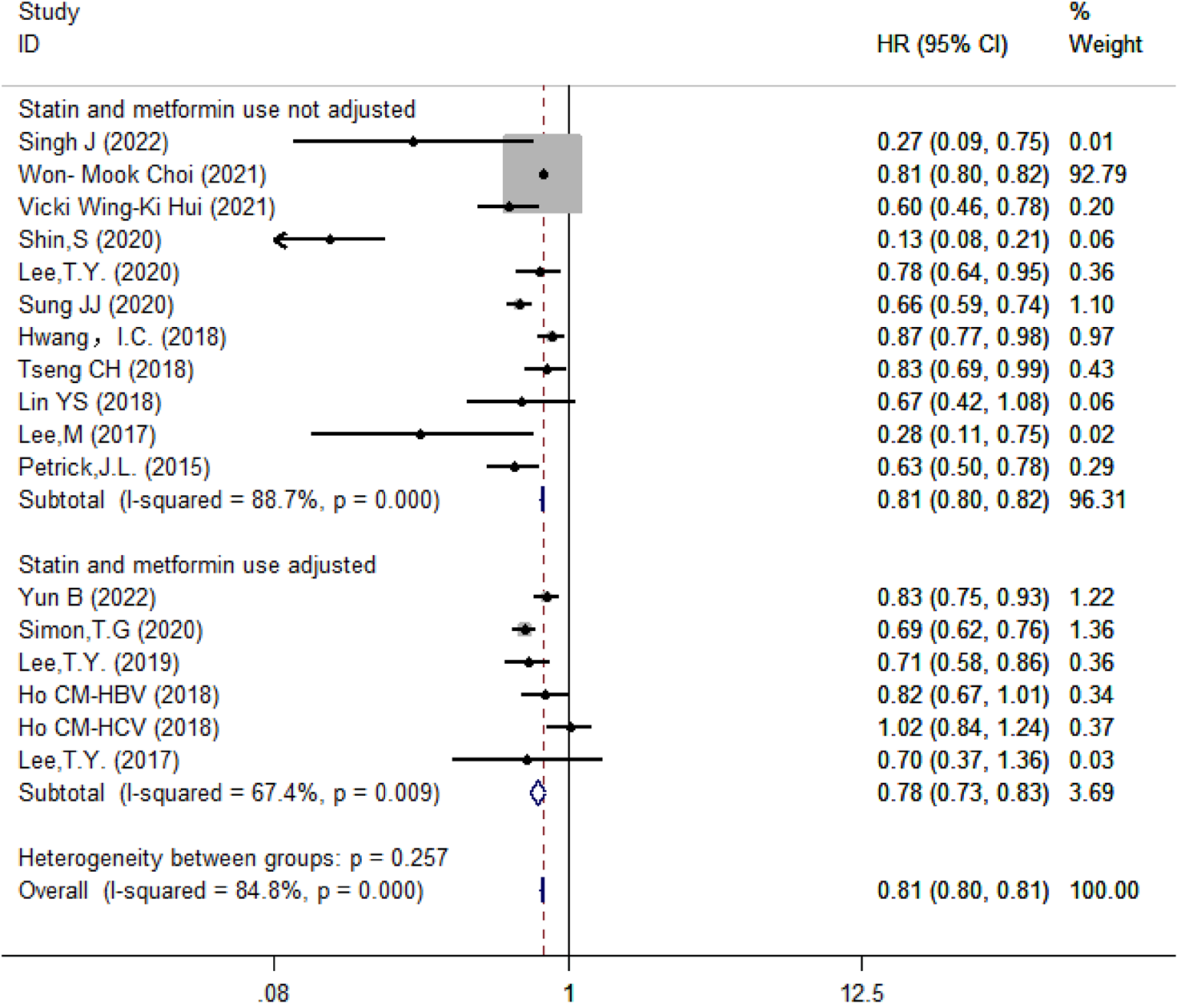
Subgroup analysis of forest plots depending on whether statins and metformin were adjusted

Then we categorized studies according to whether the results were adjusted by statins and metformin. There were 5 papers with 6 studies adjusted results for both statins and metformin and 11 papers without adjustment. The remaining two papers only adjusted results for glucose-lowering drugs, so that they were excluded. The results showed that aspirin was associated with reduced risks of HCC when the results were adjusted for statins and metformin (HR = 0.78, 95% CI 0.73–0.83, *I*^2^ = 67.4%, *P* = 0.009) or not (HR = 0.81, 95% CI 0.80–0.82, *I*^2^ = 88.7%, *P* < 0.001); however, there was no no significant difference between the two groups (*P* = 0.257) (Fig. 7).

#### Subgroup analysis based on study population

**Fig. 8:**
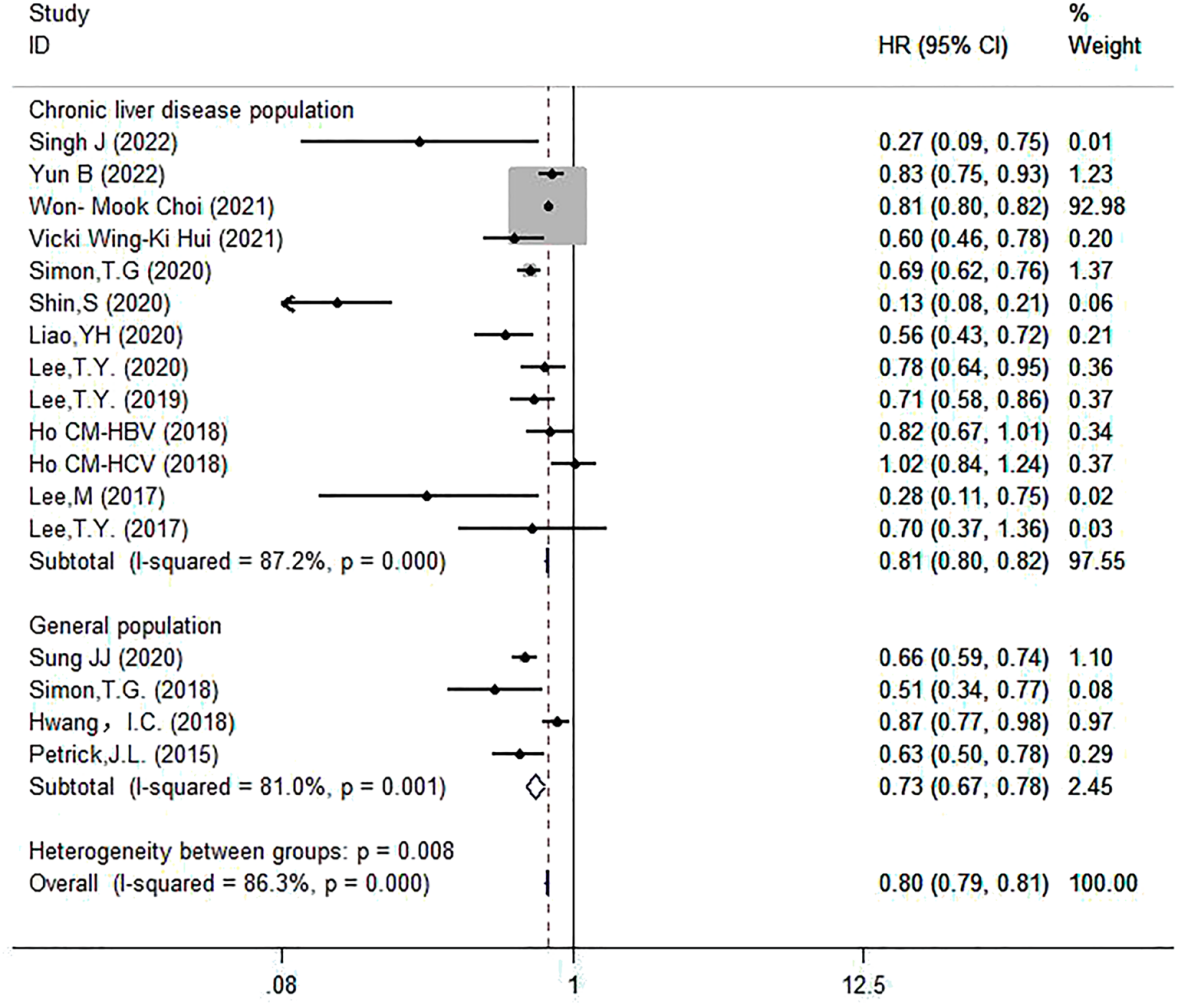
Subgroup analysis of forest plots based on the study population

Based on different populations, divided into two subgroups: patients with chronic liver disease (*n* = 13) and general population (*n* = 4). The remaining two studies were excluded. The results showed that aspirin reduced the risk of HCC in patients with chronic liver disease (HR = 0.81, 95% CI 0.80–0.82, *I*^2^ = 87.2%, *P* < 0.001) (Fig. 8).

#### Subgroup analysis based on hepatitis subtypes

**Fig. 9:**
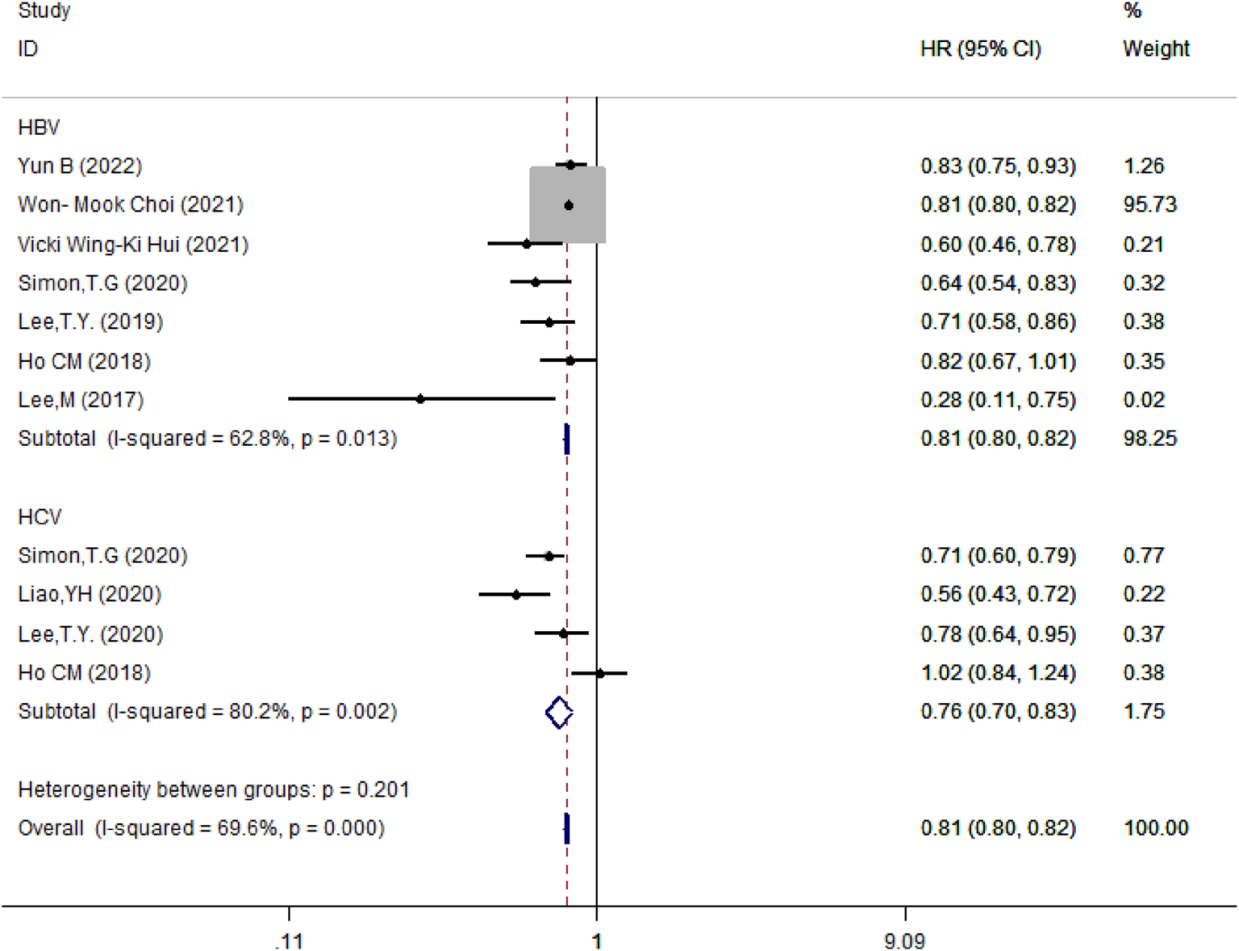
Subgroup analysis of forest plots depending on the type of infected virus

Further, in patients with liver disease, we divided studies into HBV (*n* = 7) and HCV (*n* = 4) groups based on different hepatitis viruses. The results showed that the risk of HCC was reduced by 19% in HBV-infected patients (HR = 0.81, 95% CI 0.80–0.82, *I*^2^ = 62.8%, *P* = 0.013) and by 24% in HCV-infected patients (HR = 0.76, 95% CI 0.70–0.83, *I*^2^ = 80.2%, *P* = 0.002). There was no significant difference between the two groups (*P* = 0.201) (Fig. 9).

#### Subgroup analysis based on liver cirrhosis status

**Fig. 10:**
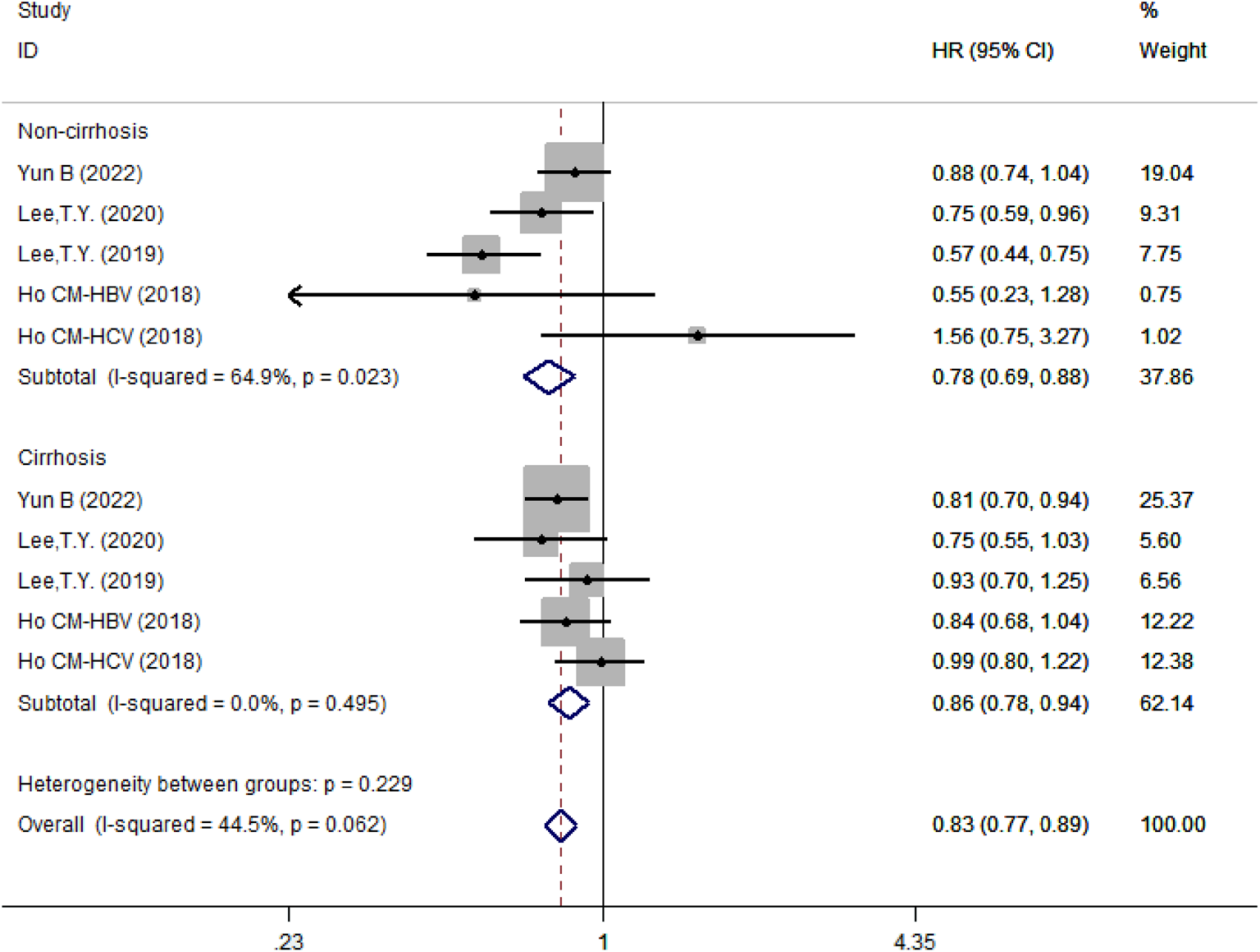
Subgroup analysis of forest plots depending on presence or absence of cirrhosis

Also, we divided patients with liver disease into liver cirrhosis and non-cirrhosis groups. The results showed that aspirin was a protective factor in either cirrhosis (HR = 0.78, 95% CI 0.69–0.88, *I*^2^ = 64.9%, *P* = 0.023) and non-cirrhosis (HR = 0.86, 95% CI 0.78–0.94, *I*^2^ = 0.0%, *P* = 0.495) groups (Fig. 10).

#### Subgroup analysis based on whether results were adjusted by antiviral drugs

**Fig. 11:**
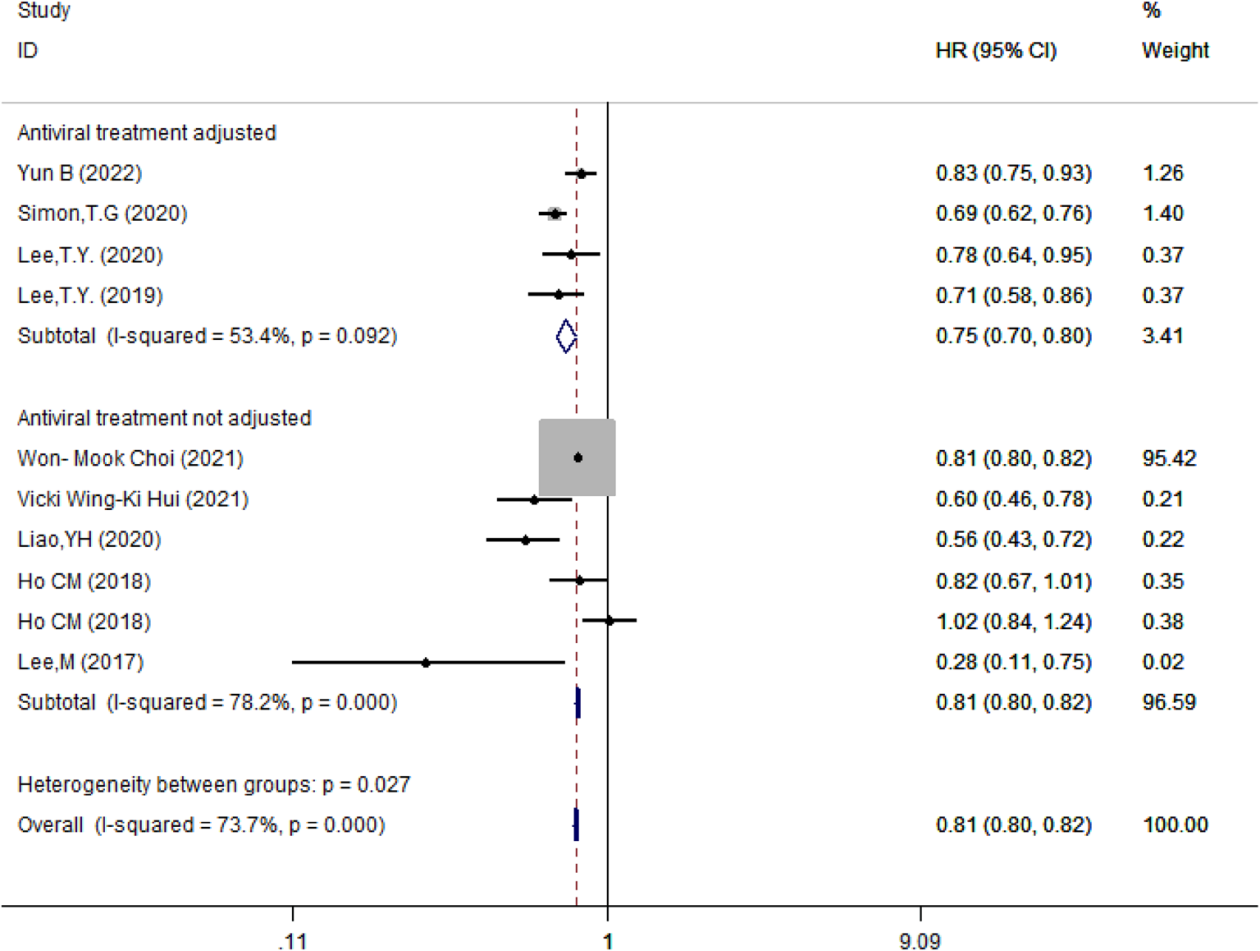
Subgroup analysis of forest plots depending on whether results were adjusted based on antiviral drugs

When studies were classified based on whether the results were adjusted by antiviral drugs, we found that aspirin remained to be a protective factor in either adjusted (HR = 0.75, 95% CI 0.70–0.80, *I*^2^ = 53.4%, *P* = 0.092) and 0.81 (95% CI 0.80–0.82, *I*^2^ = 78.2%, *P* < 0.001) respectively. There was a significant difference between groups (*P* = 0.027) (Figs. 11).

#### Analysis of bleeding risk associated with aspirin

**Fig. 12:**
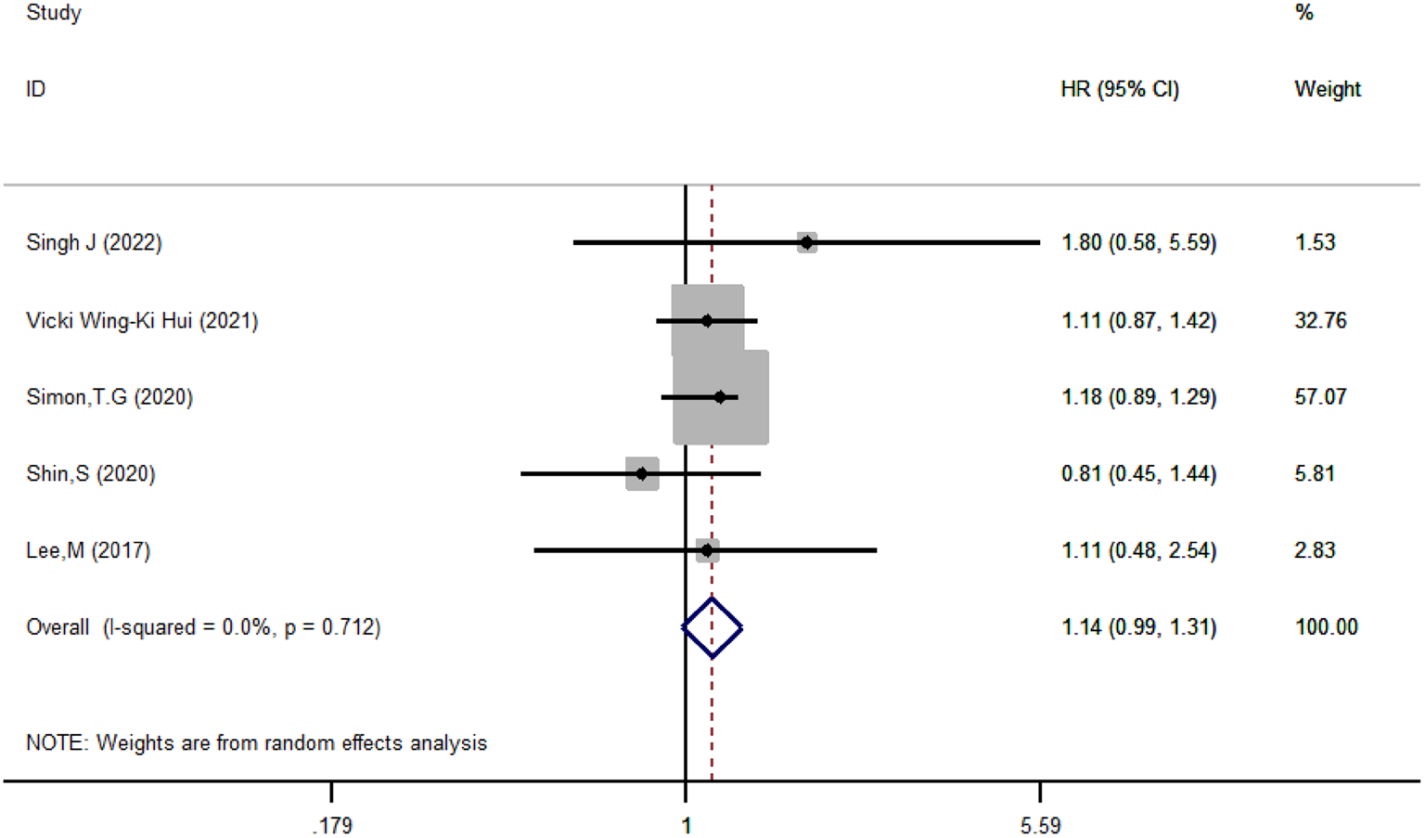
Forest plot of aspirin use and bleeding risk

To determine whether aspirin increased the risk of bleeding, we analyzed 5 papers and found that aspirin was associated with an increased risk of gastrointestinal bleeding in patients with chronic liver disease (HR = 1.14, 95% CI 0.99–1.31, *I*^2^ = 0.0%, *P* = 0.712) (Fig. 12).

#### Publication bias analysis

**Fig. 13:**
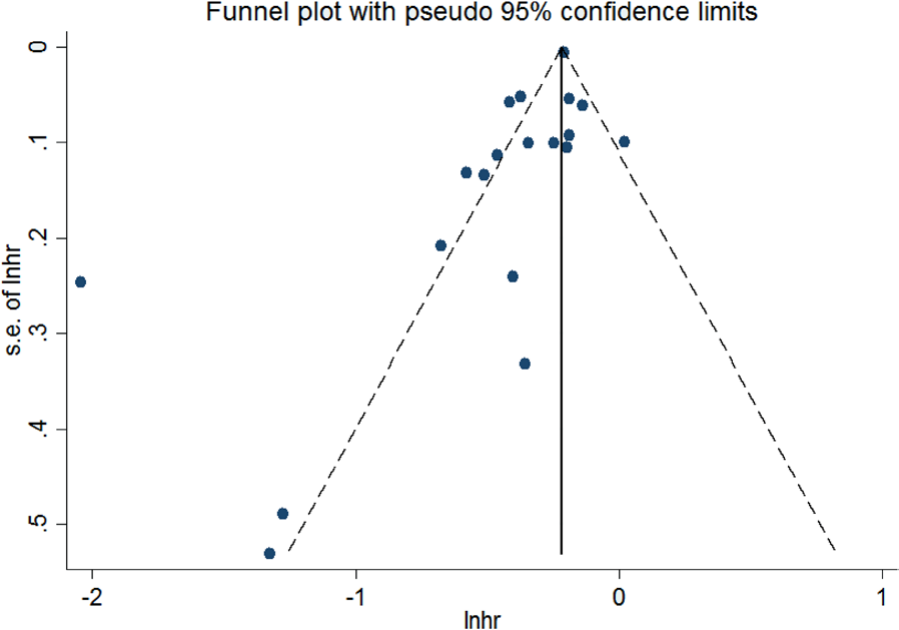
Funnel plot of the association between aspirin and the risk of hepatocellular carcinoma

Funnel plots were drawn and exhibited asymmetric, indicating the existence of publication bias. The Beggs and Eggers tests showed consistent results (Beggs: *P* = 0.093; Eggers: *P* = 0.011) (Fig. 13).

## Discussion

In recent years, many studies have demonstrated the anti-HCC effect of aspirin, which is believed to reduce the incidence of HCC [2]. However, the mechanism through which aspirin inhibits the development of HCC remains unclear. Aspirin may exert anti-HCC activity by several mechanisms [33]. For example, aspirin is able to inhibit pro-inflammatory molecule COX-2, whose inhibitor is shown to reduced liver fibrosis, portal hypertension, and hepatoma cell proliferation [37]. Also, aspirin can inhibit the activation of NF-κB pathway [34–36]. Moreover, as aspirin may induce autophagy-related cell death and interference with glucose uptake by downregulating the expression of glucose transporter protein 1 and activating Bcl-2/Bax signaling pathway [38]. In addition, aspirin can reverse sorafenib resistance in HCC and has a synergistic antitumor effect in combination with sorafenib [14].

This meta-analysis including 2,217,714 participants provides a systematic evaluation of the relationship between aspirin and the risk of HCC. Our results indicated that aspirin reduced the risk of HCC by 30% compared with that in patients who did not use aspirin. Since considerable heterogeneity was detected in the included study, we performed subgroup analyses according to study region, study type, sex, adjusted medication, and study population. Results indicated that use was associated with a reduced risk of HCC independent of these factors. As for patients with liver disease, we concluded consistent results regardless of the presence or absence of cirrhosis and adjustment for administration of antiviral drugs. In addition, the adjusted results showed a diminished effect of aspirin in reducing the risk of HCC compared with that in aspirin users who were not adjusted for both statins and metformin, suggesting that the combination of aspirin and these drugs may have a synergistic anti-HCC effect. Our results also suggest that aspirin is associated with an increased risk of gastrointestinal bleeding in patients with chronic liver disease.

Compared with previous studies, our study has a large sample size with a long follow-up period. However, there are several limitations. First, the heterogeneity of included studies was obvious, which may be due to different study designs, different and highly variable sample sizes across studies, different inclusion and exclusion criteria, the presence of more confounding factors, inconsistent control of confounding factors across studies, and varied lengths of follow-up. In addition, the presence of confounding factors in this study should be considered. For example, aspirin is often concomitantly administered with statins, lipid-lowering agents, and clopidogrel. Studies suggested that statin and clopidogrel may reduce the risk of HCC [39, 40]. Therefore, additional studies are needed to exclude the influence of confounding factors when exploring the benefit of aspirin in HCC. In addition, although aspirin has some potential benefits for patients with liver disease, its consistent use may cause some adverse events, such as gastrointestinal and intracranial hemorrhage. Therefore, it remains controversial whether aspirin should be advocated in high-risk patients such as those with peptic ulcers and esophagogastric fundic varices. Simon et al. showed that the administration of low-dose aspirin did not increase the risk of gastrointestinal bleeding [6]. Therefore, additional studies are required to determine the optimal dose and duration of aspirin to exert clinical benefit for HCC patients without increasing risks of adverse events.

## Conclusion

Aspirin may reduce the risk of HCC in both healthy population and patients with chronic liver disease. However, attention should be paid to adverse events such as gastrointestinal bleeding in patients with chronic liver disease.

## Supplementary Information

**Additional file 1.** : A combination of subject terms and free words were used for literature search using Boolean logic operator grouping.

## Data Availability

The datasets used or analyzed during the current study are available from the corresponding author on reasonable request.
