# Supplementary material for: The relationship between aspirin consumption and hepatocellular carcinoma: a systematic review and meta-analysis
Source: Eur J Med Res. 2023 Jul 8;28:226. doi: 10.1186/s40001-023-01204-5 (PMC10329378; doi:10.1186/s40001-023-01204-5)
Supplement: Supplementary file 1 — Additional file 1. : A combination of subject terms and free words were used for literature search using Boolean logic operator grouping. [file 40001_2023_1204_MOESM1_ESM.docx]

Additional file 1: A combination of subject terms and free wordswere used for literature search using Boolean logic operator grouping

| PubMed | ("Aspirin"[MeSH Terms] OR "Acetylsalicylic Acid"[All Fields] OR "acid acetylsalicylic"[All Fields] OR "2 acetyloxy benzoic acid"[All Fields] OR "Acylpyrin"[All Fields] OR "Colfarit"[All Fields] OR "Easprin"[All Fields] OR "Ecotrin"[All Fields] OR "Endosprin"[All Fields] OR "Magnecyl"[All Fields] OR "Micristin"[All Fields] OR "Polopirin"[All Fields] OR "Polopiryna"[All Fields] OR "Solprin"[All Fields] OR "Solupsan"[All Fields] OR "Zorprin"[All Fields] OR "Acetysal"[All Fields]) AND ("Liver Neoplasms"[MeSH Terms] OR "hepatocellular carcinoma "[All Fields] OR "neoplasms hepatic"[All Fields] OR "neoplasms liver"[All Fields] OR "Liver Neoplasm"[All Fields] OR "neoplasm liver"[All Fields] OR "Hepatic Neoplasms"[All Fields] OR "Hepatic Neoplasm"[All Fields] OR "neoplasm hepatic"[All Fields] OR "Cancer of Liver"[All Fields] OR "Hepatocellular Cancer"[All Fields] OR "cancers hepatocellular"[All Fields] OR "Hepatocellular Cancers"[All Fields] OR "Hepatic Cancer"[All Fields] OR "cancer hepatic"[All Fields] OR "cancers hepatic"[All Fields] OR "Hepatic Cancers"[All Fields] OR "Liver Cancer"[All Fields] OR "cancer liver"[All Fields] OR "cancers liver"[All Fields] OR "Liver Cancers"[All Fields] OR "Cancer of the Liver"[All Fields] OR "cancer hepatocellular"[All Fields]) AND ("prospective"[All Fields] OR "longitudinal"[All Fields] OR "follow-up"[All Fields] OR "cohort"[All Fields] OR "retrospective"[All Fields]) |
| --- | --- |
| Scopus | ( ( TITLE-ABS-KEY ( aspirin ) OR TITLE-ABS-KEY ( "Acetylsalicylic Acid" ) OR TITLE-ABS-KEY ( "Acid, Acetylsalicylic" ) OR TITLE-ABS-KEY ( "2-(Acetyloxy)benzoic Acid" ) OR TITLE-ABS-KEY ( acylpyrin ) OR TITLE-ABS-KEY ( aloxiprimum ) OR TITLE-ABS-KEY ( colfarit ) OR TITLE-ABS-KEY ( dispril ) OR TITLE-ABS-KEY ( easprin ) OR TITLE-ABS-KEY ( ecotrin ) OR TITLE-ABS-KEY ( endosprin ) OR TITLE-ABS-KEY ( magnecyl ) OR TITLE-ABS-KEY ( micristin ) OR TITLE-ABS-KEY ( polopirin ) OR TITLE-ABS-KEY ( polopiryna ) OR TITLE-ABS-KEY ( solprin ) OR TITLE-ABS-KEY ( solupsan ) OR TITLE-ABS-KEY ( zorprin ) OR TITLE-ABS-KEY ( acetysal ) ) ) AND ( ( TITLE-ABS-KEY ( prospective ) OR TITLE-ABS-KEY ( longitudinal ) OR TITLE-ABS-KEY ( follow-up ) OR TITLE-ABS-KEY ( cohort ) OR TITLE-ABS-KEY ( retrospective ) ) ) AND ( ( TITLE-ABS-KEY ( "Liver Neoplasms" ) OR TITLE-ABS-KEY ( "Hepatocellular Carcinoma" ) OR TITLE-ABS-KEY ( "Neoplasms, Hepatic" ) OR TITLE-ABS-KEY ( "Neoplasms, Liver" ) OR TITLE-ABS-KEY ( "Liver Neoplasm" ) OR TITLE-ABS-KEY ( "Neoplasm, Liver" ) OR TITLE-ABS-KEY ( "Hepatic Neoplasms" ) OR TITLE-ABS-KEY ( "Hepatic Neoplasm" ) OR TITLE-ABS-KEY ( "Neoplasm, Hepatic" ) OR TITLE-ABS-KEY ( "Cancer of Liver" ) OR TITLE-ABS-KEY ( "Hepatocellular Cancer" ) OR TITLE-ABS-KEY ( "Cancers, Hepatocellular" ) OR TITLE-ABS-KEY ( "Hepatocellular Cancers" ) OR TITLE-ABS-KEY ( "Hepatic Cancer" ) OR TITLE-ABS-KEY ( "Cancer, Hepatic" ) OR TITLE-ABS-KEY ( "Cancers, Hepatic" ) OR TITLE-ABS-KEY ( "Hepatic Cancers" ) OR TITLE-ABS-KEY ( "Liver Cancer" ) OR TITLE-ABS-KEY ( "Cancer, Liver" ) OR TITLE-ABS-KEY ( "Cancer, Liver" ) OR TITLE-ABS-KEY ( "Liver Cancers" ) OR TITLE-ABS-KEY ( "Cancer of the Liver" ) OR TITLE-ABS-KEY ( "Cancer, Hepatocellular" ) ) ) |
| Cochrane Library | (“Aspirin” OR "Acetylsalicylic Acid” OR "Acid, acetylsalicylic " OR "2-(Acetyloxy)benzoic Acid" OR Acylpyrin OR Aloxiprimum OR Colfarit OR Dispril OR ecotrin OR Endosprin OR Magnecyl OR Micristin OR Polopirin OR Polopiryna OR solprin OR Solupsan OR Zorprin OR Acetysal) AND (prospective OR longitudinal OR follow-up OR cohort OR retrospective) AND ("Liver Neoplasms" OR "hepatic neoplasm" OR "hepatocellular cancer" OR "hepatic cancer" OR "liver cancer" OR "liver cell carcinoma" OR "liver tumor" OR "hepatocellular carcinoma" OR HCC) |
| EMBASE | 'aspirin'/exp OR 'aspirin' OR 'acetylsalicylic acid' OR 'acid, acetylsalicylic' OR '2-(acetyloxy)benzoic acid' OR 'acylpyrin' OR 'aloxiprimum' OR 'colfarit' OR 'dispril' OR 'easprin' OR 'ecotrin' OR 'endosprin' OR 'magnecyl' OR 'micristin' OR 'polopirin' OR 'polopiryna' OR 'solprin' OR 'solupsan' OR 'zorprin' OR 'acetysal' AND 'liver neoplasms'/exp OR 'hepatocellular carcinoma' OR 'liver neoplasms' OR 'neoplasms, hepatic' OR 'neoplasms, liver' OR 'liver neoplasm' OR 'neoplasm, liver' OR 'hepatic neoplasms' OR 'hepatic neoplasm' OR 'neoplasm, hepatic' OR 'cancer of liver' OR 'hepatocellular cancer' OR 'cancers, hepatocellular' OR 'hepatocellular cancers' OR 'hepatic cancer' OR 'cancer, hepatic' OR 'cancers, hepatic' OR 'hepatic cancers' OR 'liver cancer' OR 'cancer, liver' OR 'cancers, liver' OR 'liver cancers' OR 'cancer of the liver' OR 'cancer, hepatocellular' AND 'prospective' OR 'longitudinal' OR 'follow-up' OR 'cohort' OR 'retrospective' |
| Web of Science | TS=("Liver Neoplasms" OR "Hepatocellular Carcinoma" OR "Neoplasms,Hepatic" OR "Neoplasms, Liver" OR "Liver Neoplasm" OR "Neoplasm, Liver" OR "Hepatic Neoplasms" OR "Hepatic Neoplasm" OR "Neoplasm, Hepatic" OR "Cancer of Liver" OR "Hepatocellular Cancer" OR "Cancers, Hepatocellular" OR "Hepatocellular Cancers" OR "Hepatic Cancer" OR "Cancer, Hepatic" OR "Cancers, Hepatic" OR "Hepatic Cancers" OR "Liver Cancer" OR "Cancer, Liver" OR "Cancers, Liver" OR "Liver Cancers" OR "Cancer of the Liver" OR "Cancer, Hepatocellular" OR HCC) AND TS=(Aspirin OR "Acetylsalicylic Acid" OR "Acid, Acetylsalicylic" OR "2-(Acetyloxy)benzoic Acid" OR Acylpyrin OR Aloxiprimum OR Colfarit OR Dispril OR Easprin OR Ecotrin OR Endosprin OR Magnecyl OR Micristin OR Polopirin OR Polopiryna OR Solprin OR Solupsan OR Zorprin OR Acetysal) AND TS=(prospective OR longitudinal OR follow-up OR cohort) |
